# Effect of Connection Geometry and Cantilever Length on the Mechanical Behavior of Ti6Al4V and PEEK Bar-Supported Implant Prostheses with Angled Abutments

**DOI:** 10.3390/jfb17090458

**Published:** 2026-09-08

**Authors:** Gökçen Akgün

**Affiliations:** Department of Mechanical Engineering, Faculty of Engineering, Iğdır University, 76000 Iğdır, Türkiye; gokcen.akgun@igdir.edu.tr

**Keywords:** angled abutment, implant prosthesis biomechanics, cantilever length, Ti6Al4V, PEEK, full arch prosthesis, finite element modeling

## Abstract

Angled abutments and cantilever extensions in bar-supported full-arch implant prostheses may generate bending moments and localized stress concentrations within prosthetic components and peri-implant bone. Implant-supported rehabilitation becomes biomechanically more demanding in completely edentulous patients with posterior bone resorption. Therefore, understanding how connection geometry, cantilever length, and framework material influence prosthetic design is important. In this study, a three-dimensional mandibular model generated from CT data was used to compare conventional and spherical abutment–bar connection designs in All-on-Four prostheses with 30° angled posterior abutments. Cantilever lengths of 8, 10, and 12 mm were evaluated, and Ti6Al4V Grade 5 and PEEK were assigned as alternative bar prosthesis materials. Finite element analyses were performed in ANSYS Workbench, and von Mises stress distributions were evaluated in the bar prosthesis, prosthetic screws, abutments, implants, and surrounding cortical and trabecular bone. The results showed that increasing cantilever length increased stress levels in prosthetic components and bone tissues. The spherical connection design generally reduced stresses in the most critical distal components compared with the conventional design; however, this reduction was not uniform for all components, particularly in the PEEK models. The highest stress value was observed in the posterior angled abutment of the PC-12 model with a PEEK bar prosthesis (1592.2 MPa). Among the implant components, the lowest stress was observed in the anterior implant of the TS-8 model (27.99 MPa). In contrast, the lowest bone stress was recorded in the anterior trabecular bone capsule of the PC-8 model. These findings indicate that cantilever length and connection geometry are critical parameters in designing full-arch implant prostheses. At the same time, the use of PEEK as a bar material should be interpreted with caution under extended-cantilever conditions.

## 1. Introduction

Conventional complete dentures have long been regarded as the standard clinical treatment for complete edentulism; however, developments in dental implantology have introduced implant-retained, implant-supported removable, and implant-supported fixed prosthetic systems as reliable contemporary therapeutic alternatives [1,2,3]. The long-term clinical success of osseointegrated implants has shown that implant-supported fixed prostheses may provide improved function, comfort, and patient satisfaction compared with conventional complete dentures [3,4]. Progressive alveolar bone resorption in completely edentulous patients may complicate ideal implant positioning, particularly in the posterior mandible [5,6]. Although bone augmentation procedures may be considered for insufficient bone height or width, their invasiveness, cost, healing time, and potential complications have led to the use of angled posterior implants in selected clinical situations [5,7]. In this context, the All-on-Four concept involves placing two anterior implants and two tilted posterior implants, thereby improving anteroposterior distribution and increasing prosthetic support without extensive grafting procedures [7,8].

A restricted anteroposterior implant distribution may shorten the prosthetic arch and reduce posterior functional support in edentulous patients with posterior alveolar bone loss [9,10]. This limitation may reduce the functional occlusal range, particularly in the posterior region [8,11]. To compensate, cantilever extensions are frequently incorporated into implant-supported full-arch prostheses to restore posterior function and improve masticatory efficiency [2,12]. However, loads applied at the distal cantilever region generate a lever-arm effect, increasing bending moments and stress concentrations in the prosthetic framework, abutments, screws, implants, and peri-implant bone [2,13]. Therefore, cantilever length, connection geometry, and bar framework material should be considered together rather than as independent design variables [14]. Marginal bone loss and prosthetic complications reported in fixed implant prostheses with extended cantilevers may be associated with these biomechanical effects [13,15].

The bar framework is a main load-bearing component of implant-supported prosthetic systems [16]. Ti6Al4V Grade 5 is widely used because of its high strength, stiffness, corrosion resistance, and biocompatibility; however, researchers have also investigated alternative framework materials [2,17,18]. In this regard, polyetheretherketone (PEEK) has attracted attention as a polymeric framework material because it has a lower elastic modulus and greater elastic deformation capacity than titanium alloys [2,19]. Although PEEK has shown favorable mechanical behavior in certain prosthetic applications [20], its relatively low strength compared with Ti6Al4V remains a significant mechanical limitation [21,22]. This lower stiffness may reduce stress within the framework in some configurations, but it may also increase deformation and transfer higher loads to screws, abutments, implants, and bone tissues.

Finite element analysis (FEA), based on mechanical and mathematical modeling principles, is widely used as a preliminary method for evaluating implant-supported prosthetic designs before in vitro, in vivo, or clinical validation [23,24]. FEA allows comparison of different prosthetic configurations, material properties, loading conditions, and connection designs under controlled conditions [2,5]. Although previous finite element studies have investigated the biomechanical behavior of All-on-Four prostheses with tilted implants, the combined influence of abutment–bar connection geometry, cantilever length, and bar material on stress distribution remains insufficiently clarified. Therefore, the present study evaluated conventional and spherical abutment–bar connection designs in full-arch implant-supported prostheses with 30° angled posterior abutments. The novelty of the study lies in assessing whether a spherical connection interface can reduce stress concentrations under different cantilever lengths and bar materials, including Ti6Al4V Grade 5 and PEEK.

## 2. Materials and Methods

**Figure 1:**
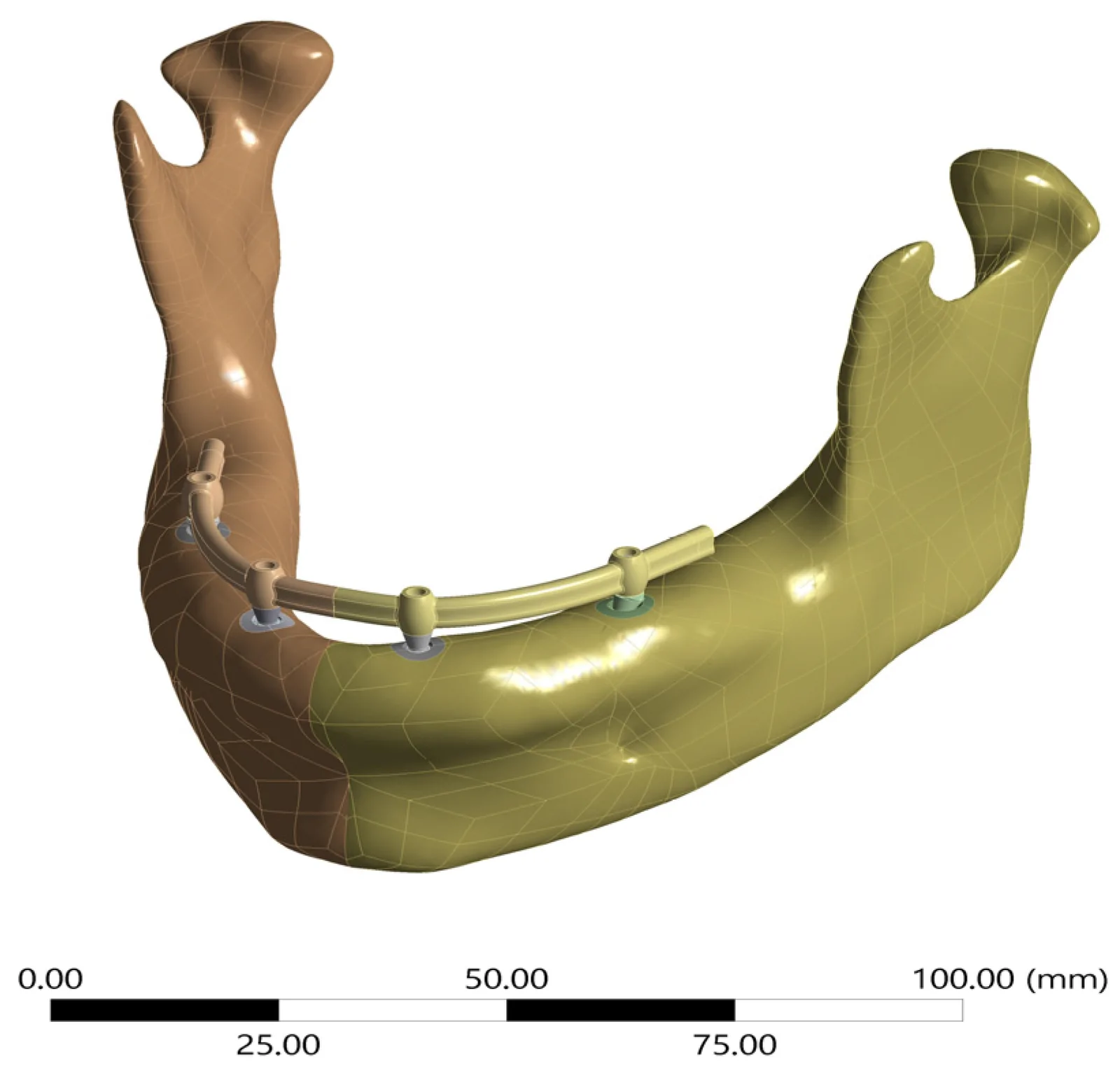
3D solid model design of the complete mandibular bone model together with the dental prosthesis (conventional model) with a 10 mm cantilever length.

In this study, we evaluated the stress and strain behavior of implant-supported prosthetic components using FEA in ANSYS Workbench 2019 R3 (ANSYS Inc., Canonsburg, PA, USA). Mandibular CT data (from Embodi3D (https://www.embodi3d.com/files/file/17939-mandibula-stl-file-processed/ (accessed on 15 May 2022))) were initially processed in Mimics 27.0 (Materialise, Leuven, Belgium), and the three-dimensional mandibular model was subsequently generated using SolidWorks 2022 (Dassault Systèmes SolidWorks Corp., Waltham, MA, USA). The CT-based geometry was used to construct a representative edentulous mandibular model; however, this analysis did not account for patient-specific anatomical variability. Except for the modified bar-connection design, the implant and abutment geometries were developed from a commercially available reference design. Figure 1 shows the complete mandibular model and the assembled conventional prosthetic configuration with a 10 mm cantilever length. This figure is intended to demonstrate the overall prosthesis–mandible relationship and the position of the full-arch bar prosthesis before the comparative finite element analyses.

Two main connection configurations were developed: conventional and spherical. In the spherical design, a modified spherical interface was introduced at the abutment–bar connection region to alter load transfer and reduce local stress concentration. Sub-models were then created by varying cantilever length as 8, 10, and 12 mm. The models were classified as conventional (C-8, C-10, and C-12) and spherical (S-8, S-10, and S-12). In addition, Ti6Al4V Grade 5 and PEEK were designated as alternative bar-prosthesis materials. Accordingly, TC and TS indicate Ti6Al4V conventional and Ti6Al4V spherical models, respectively, whereas PC and PS indicate PEEK conventional and PEEK spherical models, respectively. PEEK was considered as a polymeric bar framework material, not as an alloy. The All-on-Four treatment concept was used as the reference configuration. The anterior implants were positioned in the lateral incisor region, while the distal implants were positioned between the first and second molar regions.

Due to sagittal symmetry, all models were constructed as half-geometry. We applied refined meshing around the implant–bone interface and critical prosthetic components, including the abutments, prosthetic screws, and implants. The surrounding bone tissue was modeled as cortical and trabecular capsule-like regions around the implant to allow local stress evaluation. All models used higher-order 10-node three-dimensional tetrahedral elements [25,26]. Mesh quality was assessed using the “Mesh Metric” and “Check Mesh Quality” tools in ANSYS Workbench [27]. The node and element numbers were similar across the conventional and spherical models, indicating comparable discretization among the analyzed configurations.

Table 1 summarizes the numbers of nodes and elements, the average element quality, and the skewness values for each model. These values show that similar mesh densities were maintained between the conventional and spherical designs. The mechanical properties assigned to Ti6Al4V Grade 5, PEEK, cortical bone, and trabecular bone are presented in Table 2. All materials were assumed to be homogeneous, isotropic, and linearly elastic, a common simplification in dental implant FEA that does not fully capture the anisotropic and heterogeneous behavior of bone tissue [27,28,29]. We used the same material properties across all corresponding model groups to isolate the effects of connection design, cantilever length, and bar material.

Table 2 lists the elastic modulus and Poisson’s ratio values used in the finite element models. These material inputs were held constant within each material group to compare the effects of geometry and cantilever length under controlled conditions.

Complete osseointegration was assumed at the implant–bone interfaces, and bonded contact was defined accordingly [5,29]. Bonded contact was also assigned to interfaces where relative motion was not expected. To simulate a simplified average occlusal loading condition, a vertical static load equivalent to 100 N in the full model was applied to the distal end region of the prosthetic bar, consistent with previous finite element studies evaluating mandibular implant-supported prosthetic systems [30]. Since a half-model was used because of sagittal symmetry, the applied load and symmetry constraints were defined to represent the corresponding full-model loading condition. The displacement constraints were assigned to prevent rigid body motion while preserving the intended load transfer through the prosthetic structure. This loading condition represents a simplified static scenario and does not reproduce the complex multidirectional and cyclic nature of mastication.

According to the literature, element quality values ranging from 0.70 to 0.95 are considered indicative of very good mesh quality [31]. Therefore, the mesh quality values obtained from the present models were considered acceptable for comparative finite-element evaluation. Mesh independence of the reported peak stress values was subsequently confirmed through a dedicated convergence analysis, presented in the Results Section. Frictional contact was defined at the bar prosthesis–prosthetic screw and bar prosthesis–abutment interfaces to account for potential micromovement under loading. For titanium alloy contact surfaces, we adopted a friction coefficient of 0.2, consistent with values commonly reported in the literature [27,28,29]. For PEEK–Ti6Al4V contact, we used a friction coefficient of 0.1, based on previous tribological findings for PEEK/Ti6Al4V systems in artificial saliva, where friction coefficients ranged from 0.09 to 0.14 [32].

**Figure 2:**
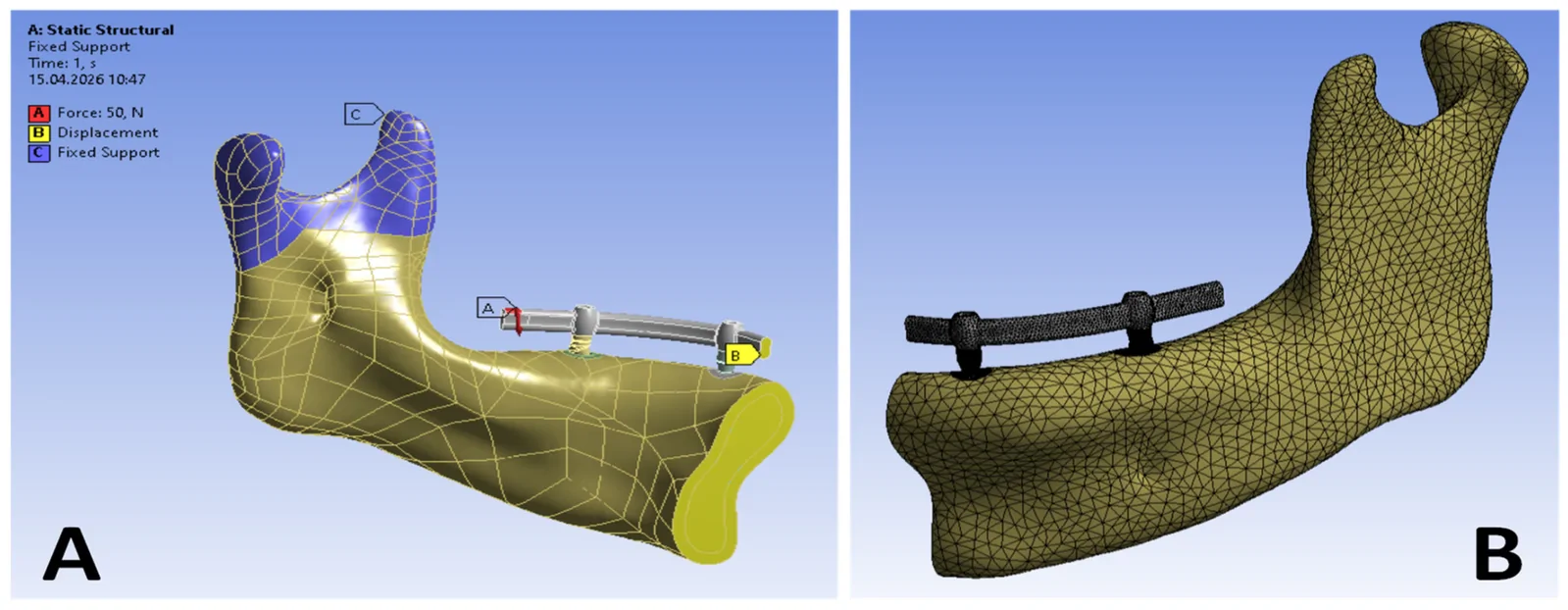
(**A**): Finite element model with illustration of boundary conditions, loading region, supports, and displacement constraints; (**B**): meshed representation of the model.

Screw preload was not incorporated into the present model, and this simplification should be considered when interpreting the absolute stress values obtained in the prosthetic screws. Preload generates a baseline clamping force at the screw–abutment and screw–bar interfaces, which increases the initial static stress state of the screw and alters the local contact pressure and frictional behavior before functional loading is applied [33]. Consequently, the absolute stress magnitudes reported for the prosthetic screws in the present study may differ from those of a pre-tightened clinical joint, with the direction and magnitude of this difference depending on preload level and loading direction [34,35]. However, because the absence of preload was applied uniformly across all conventional and spherical models, and because the connection geometry, rather than the joint pretension state, was the primary variable under investigation, the relative comparison between the two connection designs is expected to remain directionally valid. However, preload may disproportionately affect components already identified as sensitive to contact-mechanics assumptions in this study, such as the posterior bar prosthesis screw, where a non-monotonic stress response to the spherical design was observed. Future finite element models incorporating pretension bolt elements or torque-controlled preload boundary conditions are needed to confirm whether the present findings remain valid under clinically representative joint-tightening conditions [34]. Figure 2 presents the finite element model, boundary conditions, loading region, and mesh structure. Since the same boundary and meshing strategy was applied to all model groups, only a representative spherical model is shown.

**Figure 3:**
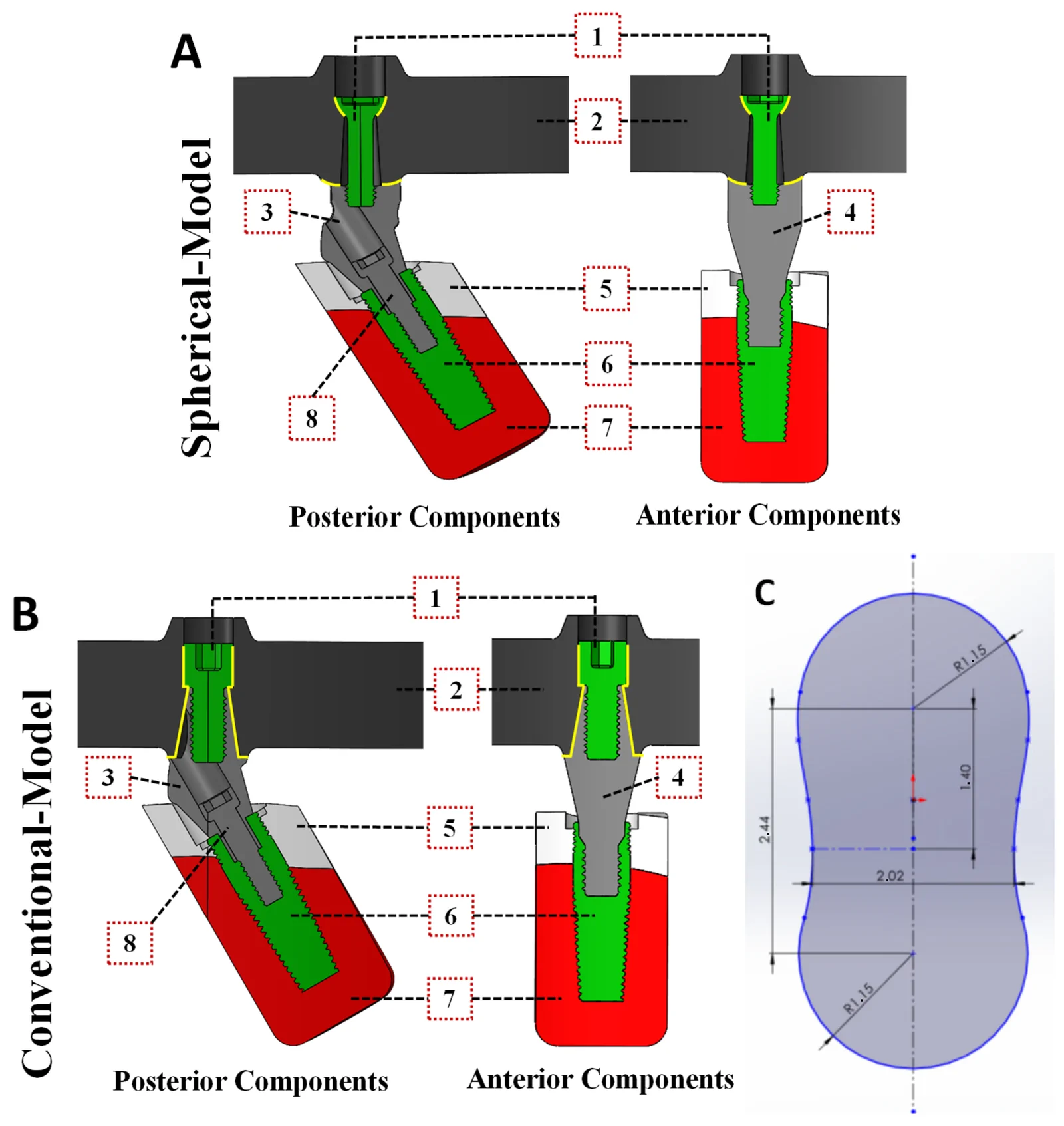
Frictional contact interfaces and prosthetic components in the spherical (**A**) and conventional (**B**) models, and cross-sectional design of the dental prosthetic bar (**C**). The numbered components correspond to the following: (1) bar prosthetic screw, (2) bar prosthesis, (3) posterior angled abutment, (4) anterior abutment, (5) cortical bone capsule, (6) dental implant, (7) trabecular bone capsule, and (8) abutment–implant connecting screw.

Cantilever length is a main geometric parameter affecting stress distribution in implant-supported prostheses, and clinically used cantilever lengths may vary depending on anatomical and prosthetic limitations [2,36]. In the present study, cantilever lengths of 8, 10, and 12 mm were selected to represent short, intermediate, and extended distal extension conditions. The prosthetic components and frictional contact interfaces were identified to clarify how load is transferred between the bar, screws, abutments, implants, and the surrounding bone. The elastic modulus and Poisson’s ratio of PEEK were selected in accordance with previous finite element studies comparing PEEK with metallic framework materials in dental prosthetic applications [37]. Figure 3 illustrates the frictional contact interfaces and the main prosthetic components in the spherical and conventional connection designs. The red regions indicate the interfaces where frictional contact was defined, while the numbered components identify the main structural parts included in the finite element model. From a theoretical standpoint, the distal extension of the bar prosthesis can be idealized as a cantilever beam loaded at its free end [38]. According to Bernoulli–Euler beam theory, the bending moment at the fixed support is expressed as

M = F × L(1)

and the bending stress at the outermost fiber is given by

σ = M·c/I = (F·L·c)/I(2)

where F is the applied occlusal force, L is the cantilever length, c is the distance from the neutral axis to the outermost fiber, and I is the second moment of area of the cross-section. This relationship predicts that, under the same load and cross-sectional geometry, bending stress increases with cantilever length. The governing differential equation for elastic beam bending is expressed as

E.I.d^2^y/dx^2^ = −M (3)

where x denotes the distance along the beam, y is the transverse deflection, E is Young’s modulus, I is the second moment of area, and M is the internal bending moment [39]. Although the prosthetic bar is geometrically more complex than an ideal beam, this formulation provides a mechanical explanation for the increase in stress observed with increasing cantilever length.

## 3. Results

In this study, we evaluated von Mises stress distributions in prosthetic components and surrounding bone tissues for conventional and spherical connection designs with cantilever lengths of 8, 10, and 12 mm. Table 3 and Table 4 present the stress values obtained for the Ti6Al4V and PEEK bar prosthesis models, respectively. In general, increasing cantilever length increased stress levels in the bar prosthesis, prosthetic components, implants, and bone tissues. This trend is consistent with the theoretical expectation that increasing cantilever length increases the bending moment acting on the prosthetic system [38,39]. The lowest stress levels were generally associated with the 8 mm cantilever models, whereas the 12 mm cantilever models produced the highest stresses. Because no statistical test was performed, the differences between models are reported numerically rather than as statistically significant.

**Figure 4:**
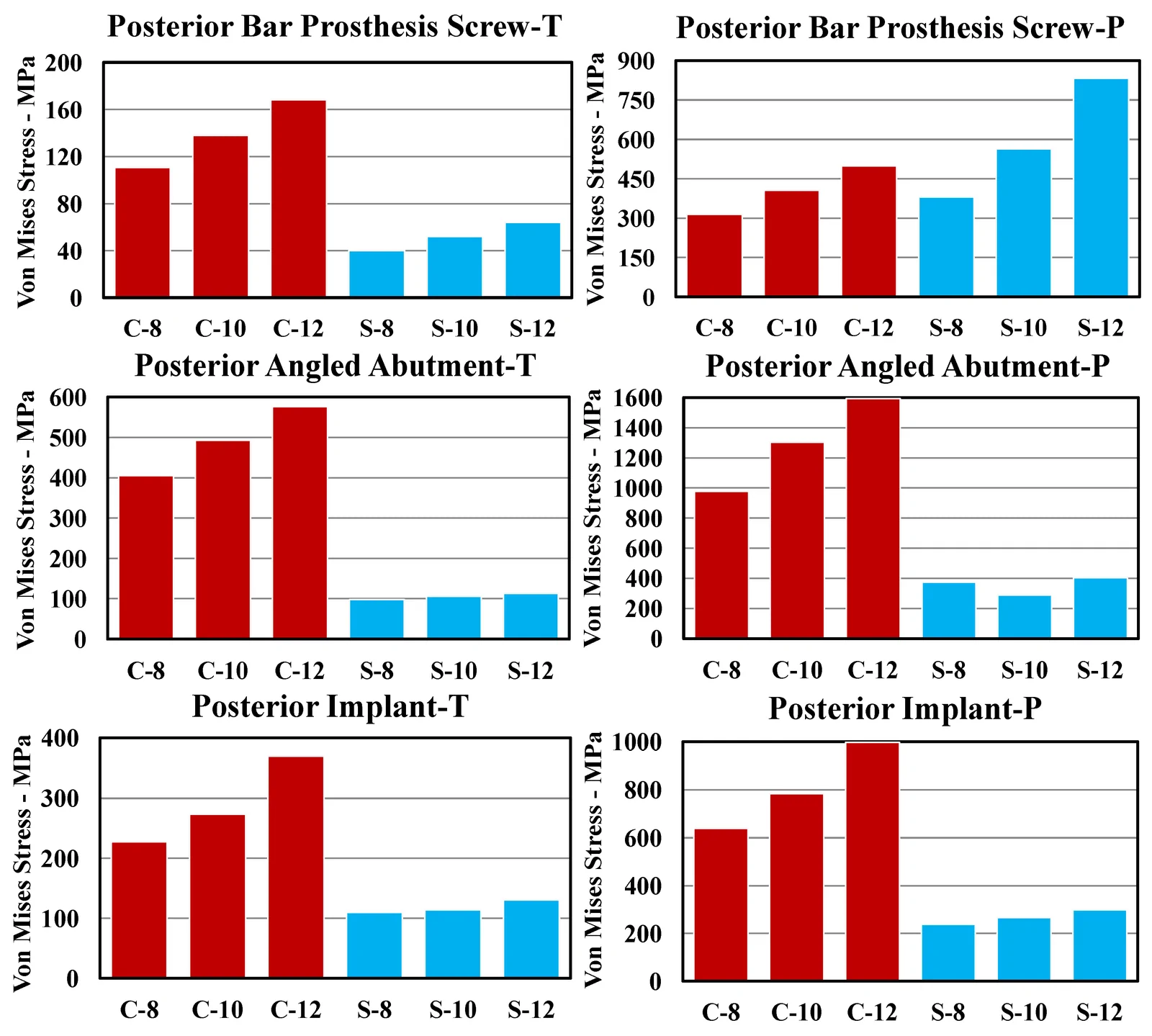
Comparative bar charts of von Mises stress values (MPa) for the most critical prosthetic components in the two different material bar prosthesis models (T: Ti6Al4V; P: PEEK), corresponding to the data presented in Table 3 and Table 4. Red bars represent the conventional connection design (C-8, C-10, C-12) and blue bars represent the spherical connection design (S-8, S-10, S-12).

**Figure 5:**
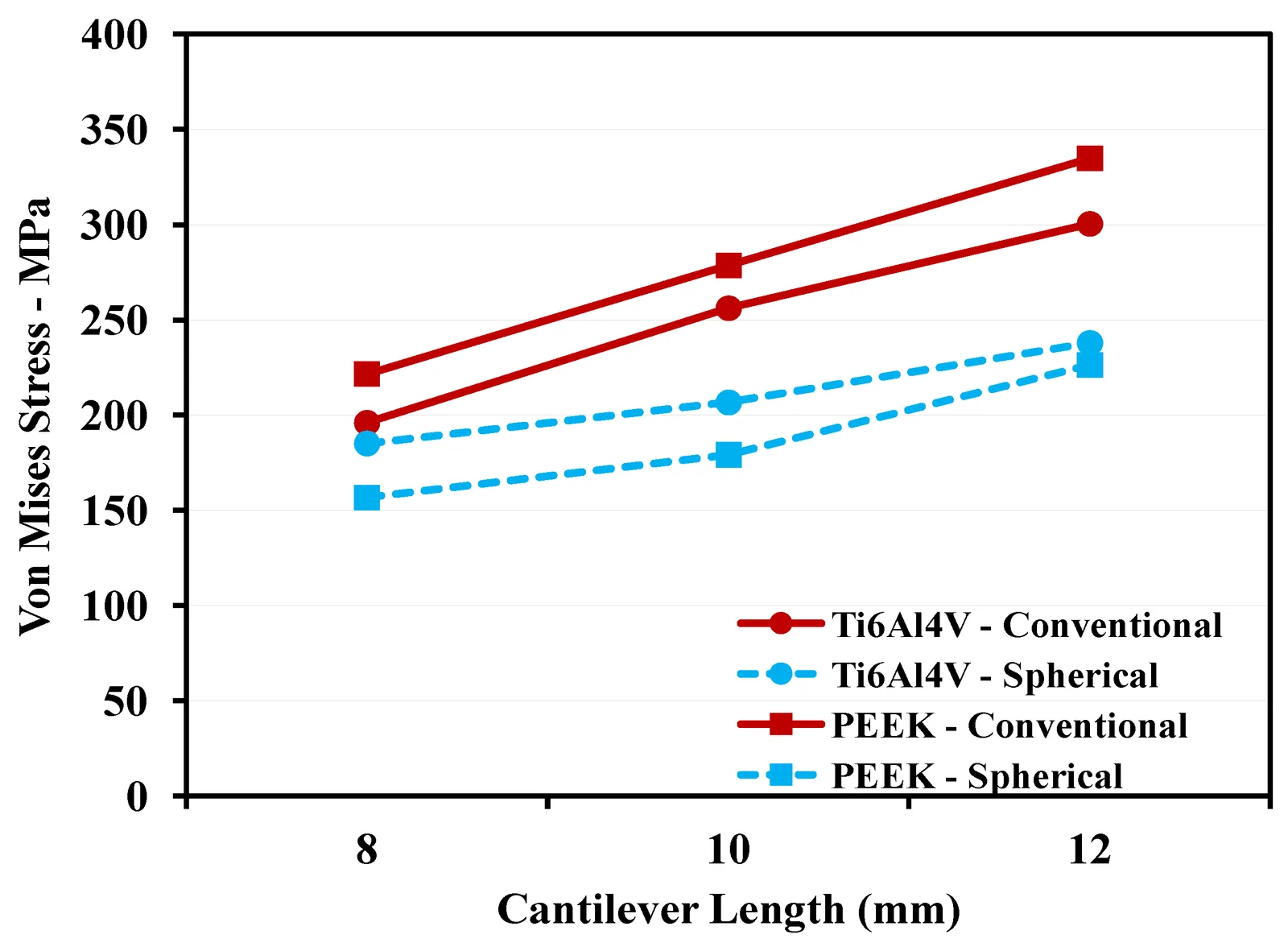
Von Mises stress in the bar prosthesis versus cantilever length (8, 10, 12 mm) for Ti6Al4V and PEEK under conventional and spherical connection designs.

Table 3 presents the von Mises stress values for the models in which Ti6Al4V Grade 5 was assigned as the bar prosthesis material. The table allows a direct comparison of conventional and spherical connection designs for cantilever lengths of 8, 10, and 12 mm. Furthermore, due to the bending moment arising from their proximity to the distal cantilever region, the stress variations in the bar prosthesis screw, angled abutment, and implant, which represent the most critical prosthetic components, are also presented graphically as a comparison across the different bar prosthesis materials (Figure 4). For the bar prosthesis component, the effect of cantilever length on stress distribution is likewise presented comparatively for the two bar prosthesis materials in Figure 5.

Table 4 presents the von Mises stress values for the models in which PEEK was assigned as the bar prosthesis material. These results show how PEEK’s lower stiffness affected stress transfer to prosthetic screws, abutments, implants, and bone tissues under the same geometric conditions.

For the Ti6Al4V bar prosthesis models, the spherical connection design reduced stress values in the most critical posterior components compared with the conventional design. In the 12 mm cantilever group, the spherical design reduced posterior implant stress from 369.95 MPa to 130.95 MPa, corresponding to a 64.6% reduction. Similarly, stress in the posterior angled abutment decreased from 576.97 MPa to 113.30 MPa, corresponding to an 80.36% reduction. Reductions of 20.82%, 29.11%, and 4.5% were also observed in the bar prosthesis, posterior cortical bone capsule, and posterior trabecular bone capsule, respectively. These results indicate that the spherical connection design was most effective in reducing stress in distal prosthetic components, especially the posterior angled abutment and posterior implant.

For the PEEK bar prosthesis models, the effect of spherical connection geometry was more component-dependent. In the 12 mm cantilever group, the spherical design reduced posterior implant stress from 996.71 MPa to 298.99 MPa, corresponding to a 70.0% reduction. Stress in the posterior angled abutment decreased from 1592.20 MPa to 403.82 MPa, corresponding to a 74.63% reduction. Reductions of 32.39%, 44.55%, and 39.75% were also observed in the bar prosthesis, posterior cortical bone capsule, and posterior trabecular bone capsule, respectively. However, some anterior components, particularly the anterior bar prosthesis screw and anterior abutment, showed higher stress values in the spherical PEEK models than in the corresponding conventional PEEK models. Therefore, the spherical design should not be described as reducing stress in all components under all conditions.

**Figure 6:**
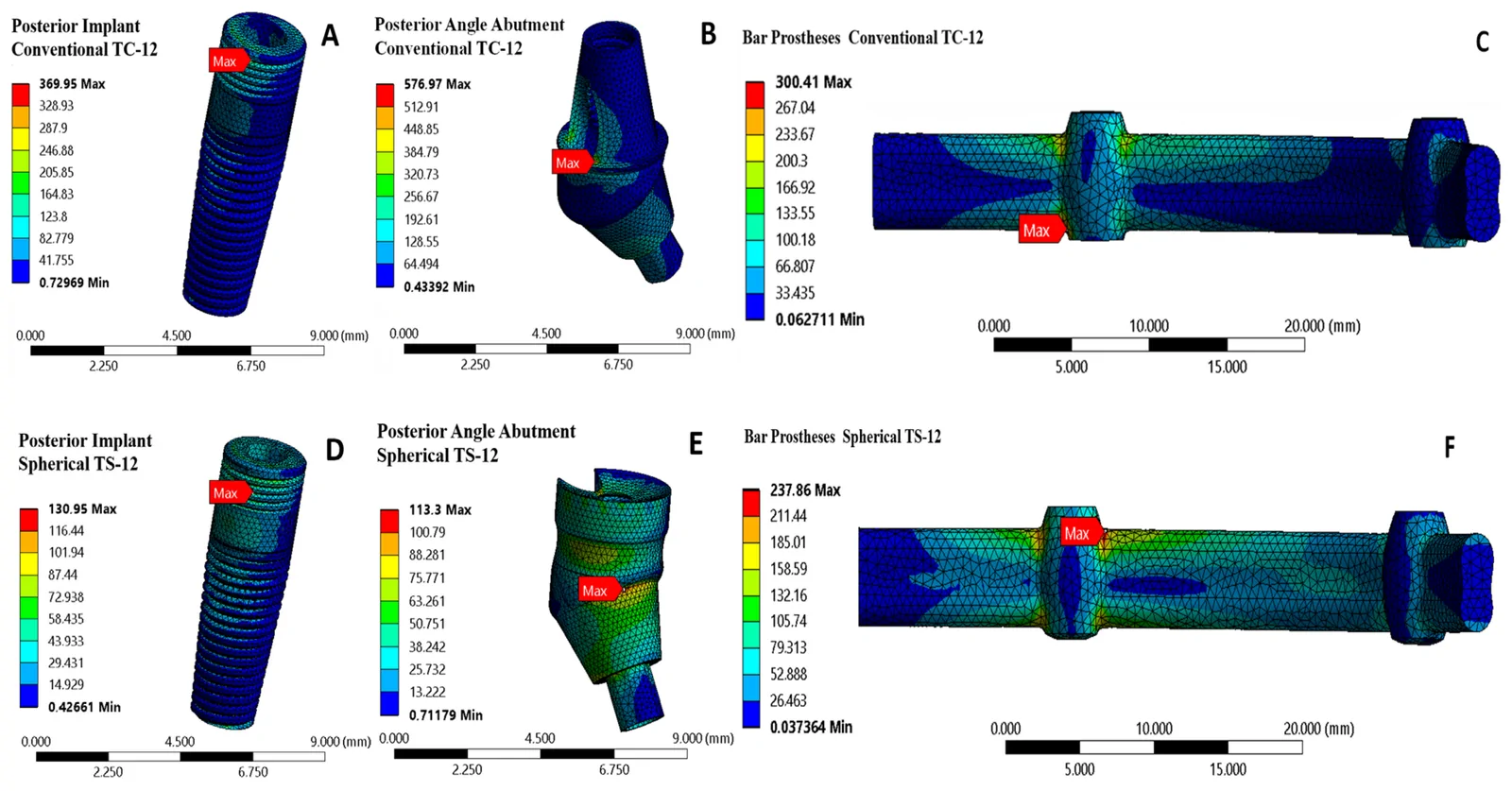
Variation in stress (MPa) patterns of the bar prosthesis (Ti6Al4V), angled abutment, and distal implant components in the conventional (**A**–**C**) and spherical (**D**–**F**) design models.

**Figure 7:**
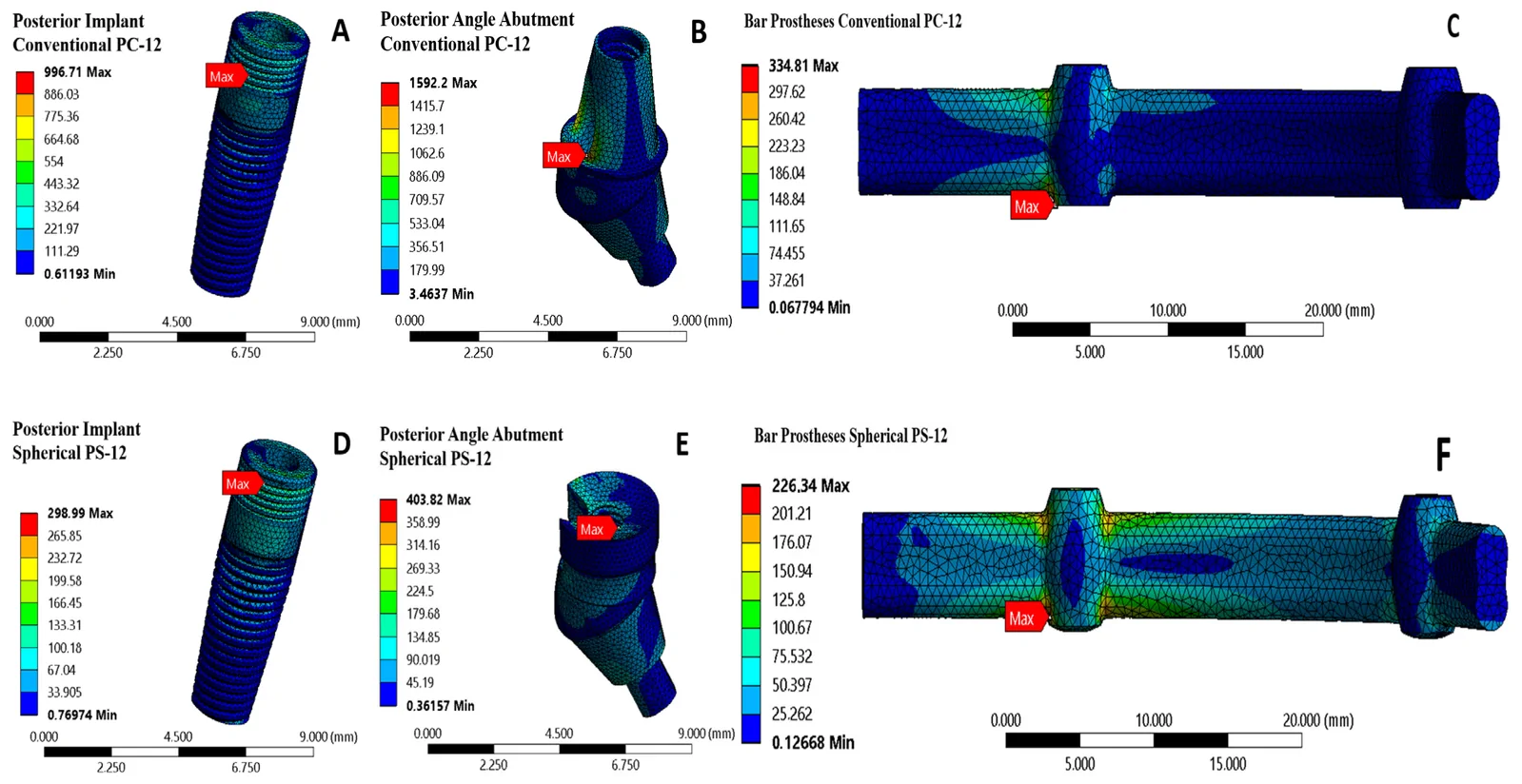
Variation in stress (MPa) patterns of the bar prosthesis (PEEK), angled abutment, and distal implant components in the conventional (**A**–**C**) and spherical (**D**–**F**) design models.

The stress distributions of the most critical distal components, including the bar prosthesis, posterior angled abutment, and posterior implant, are shown in Figure 6 and Figure 7 for the 12 mm cantilever models. These figures visually corroborate the numerical results in Table 3 and Table 4, showing that stress concentrations were primarily localized in the distal prosthetic region. Figure 6 shows the stress distribution patterns in the Ti6Al4V bar prosthesis models with a 12 mm cantilever length. The comparison between conventional and spherical designs highlights reduced stress concentration around the posterior angled abutment and posterior implant in the spherical configuration.

Figure 7 shows the stress distribution patterns in the PEEK bar prosthesis models with a 12 mm cantilever length. The spherical design reduced stress concentrations in the posterior implant and posterior-angled abutment compared with the conventional PEEK model, although stress redistribution to other components should be considered.

**Figure 8:**
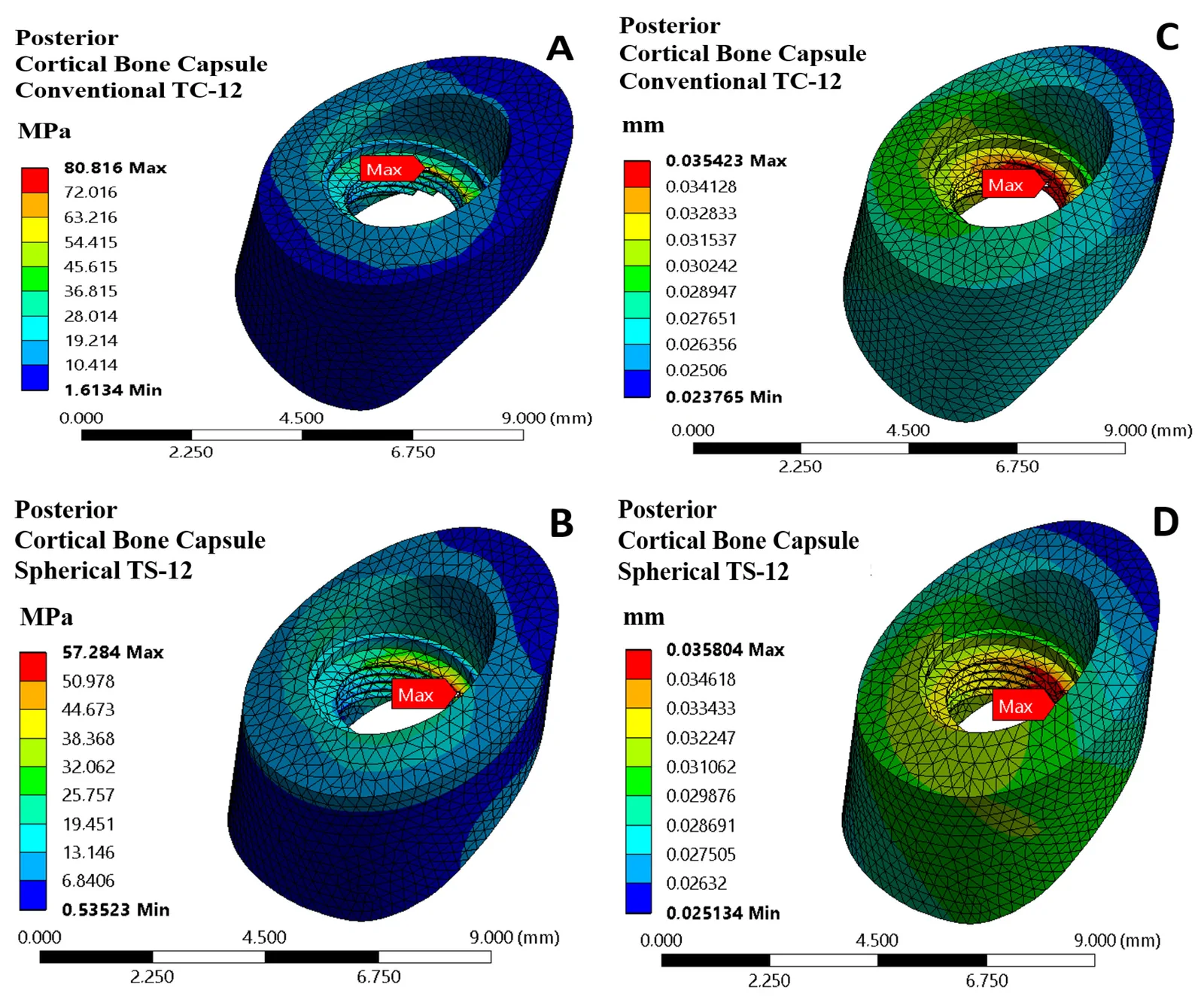
Von Mises stress (**A**,**B**) and deformation (**C**,**D**) variations in the posterior cortical bone tissues in the conventional TC-12 and spherical TS-12 models. Bar prosthesis material: Ti6Al4V.

**Figure 9:**
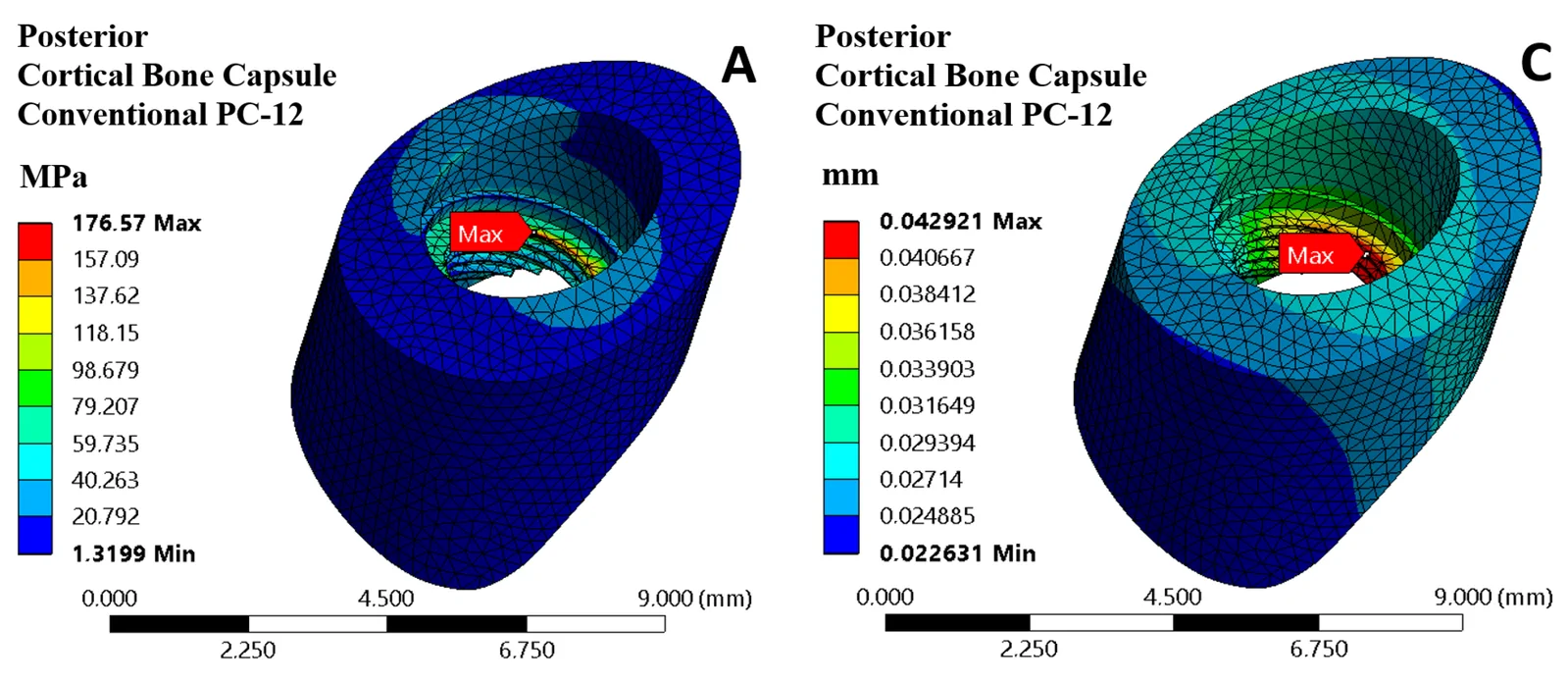

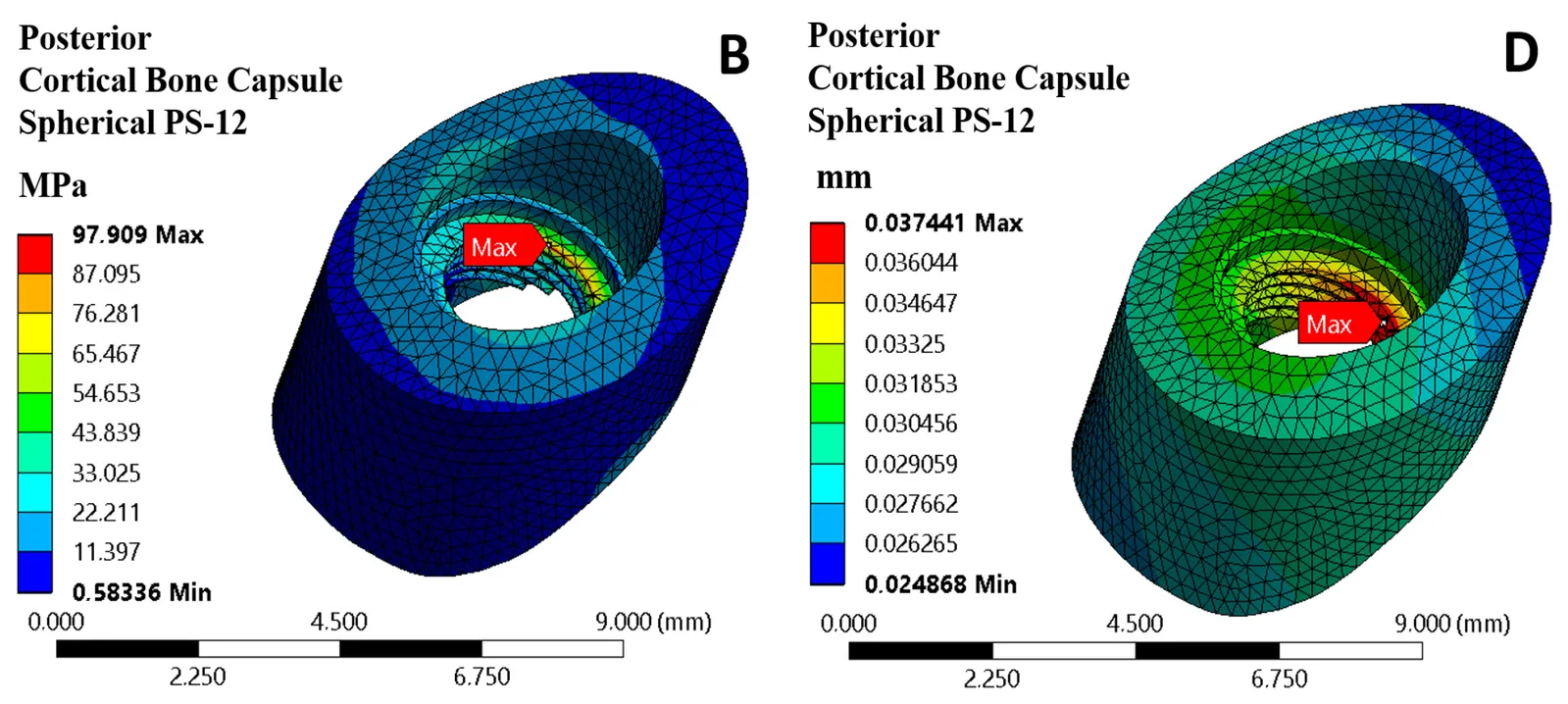
Von Mises stress (**A**,**B**) and deformation (**C**,**D**) variations in the posterior cortical bone tissues in the conventional PC-12 and spherical PS-12 models. Bar prosthesis material: PEEK.

**Figure 10:**
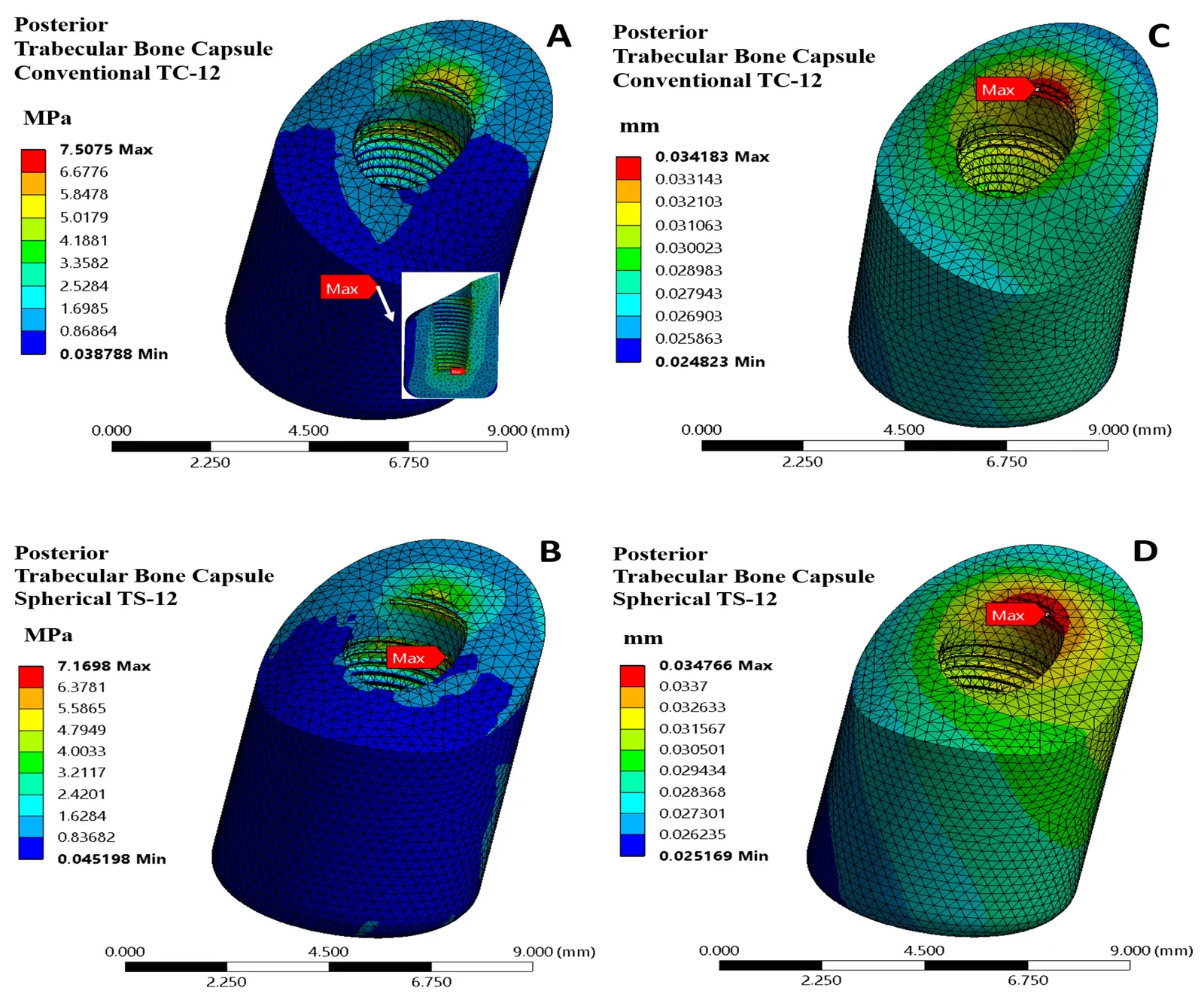
Von Mises stress (**A**,**B**) and deformation (**C**,**D**) variations in the posterior trabecular bone tissues in the conventional TC-12 and spherical TS-12 models. Bar prosthesis material: Ti6Al4V.

**Figure 11:**
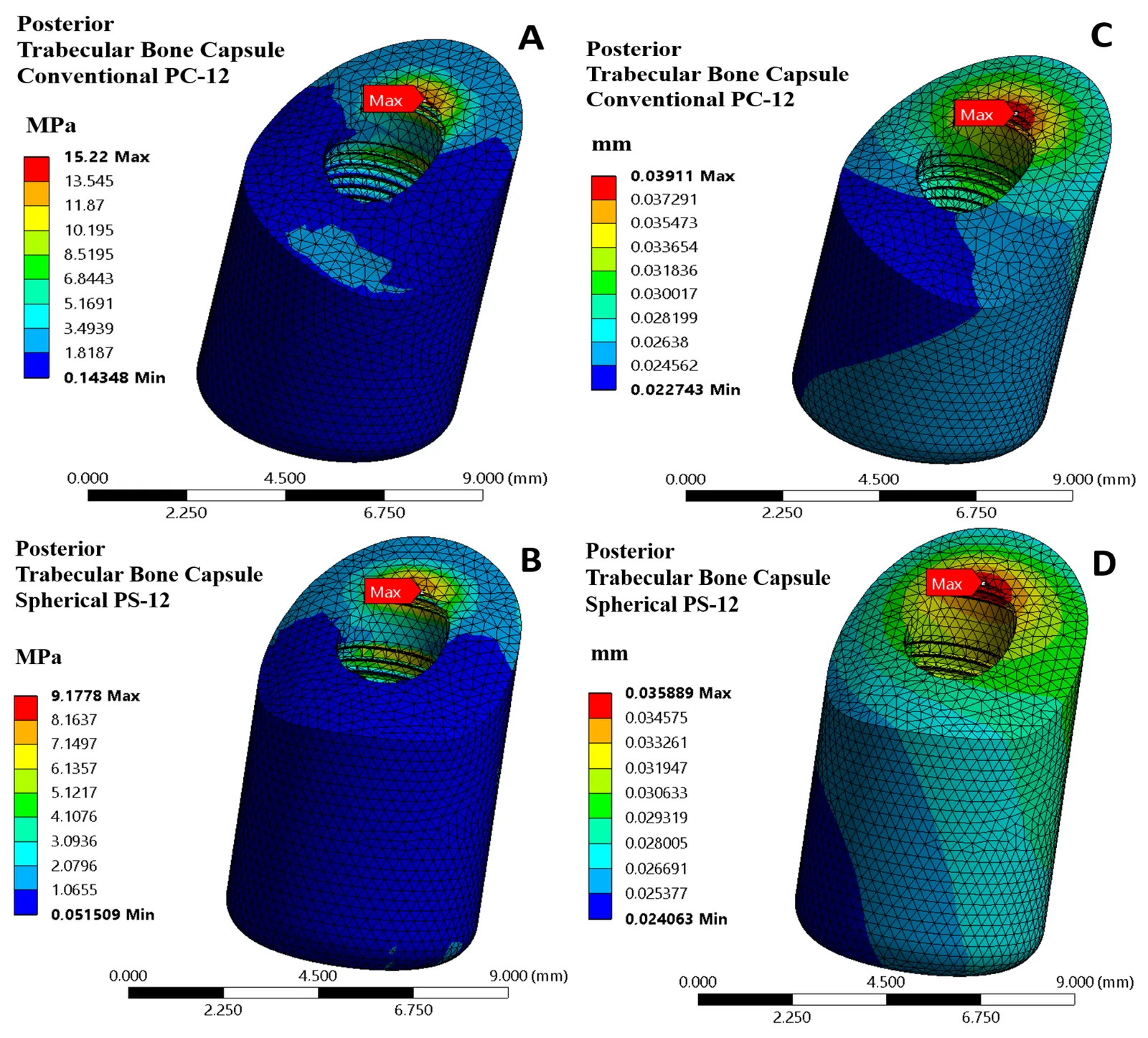
Von Mises stress (**A**,**B**) and deformation (**C**,**D**) variations in the posterior trabecular bone tissues in the conventional PC-12 and spherical PS-12 models. Bar prosthesis material: PEEK.

Figure 8, Figure 9, Figure 10 and Figure 11 present the stress and deformation distributions in the posterior cortical and trabecular bone tissues for the 12 mm cantilever configurations most critical to the analysis. These figures clarify how connection geometry and bar material affect peri-implant bone response. In general, spherical models showed lower posterior bone stress and deformation than conventional models, particularly in the PEEK group; however, these findings should be interpreted in light of the assumptions of static loading and simplified bone material behavior. Figure 8 compares von Mises stress and deformation in the posterior cortical bone for the conventional TC-12 and spherical TS-12 models. The figure demonstrates the effect of spherical connection geometry on cortical bone response when Ti6Al4V is used as the bar prosthesis material.

Figure 9 compares von Mises stress and deformation in the posterior cortical bone for the conventional PC-12 and spherical PS-12 models. The figure shows that the spherical connection design reduced cortical bone stress in the PEEK bar prosthesis group.

Figure 10 shows von Mises stress and deformation distributions in the posterior trabecular bone for the conventional TC-12 and spherical TS-12 models. This comparison illustrates the trabecular bone response under a Ti6Al4V bar prosthesis.

Figure 11 shows von Mises stress and deformation distributions in the posterior trabecular bone for the conventional PC-12 and spherical PS-12 models. This comparison illustrates the trabecular bone response under the PEEK bar prosthesis condition.

To verify the numerical stability of the highest stress value reported in this study, we performed a mesh convergence analysis at the posterior angled abutment interface of the PC-12 model, which exhibited the highest von Mises stress (1592 MPa) among all evaluated configurations. Starting from the mesh density used in the reported results (element size = 0.18 mm), the local mesh was progressively refined in four additional steps down to an element size of 0.14 mm (Table 5). The percentage change in maximum von Mises stress between consecutive refinement levels fell below the 5% convergence threshold from the second refinement level onward (2.32%, 1.10%, 0.67%, and 0.36%, respectively). The overall difference between the production mesh used throughout the study and the finest tested mesh was 4.52%, confirming that the reported mesh density provided a numerically stable estimate of the peak stress value. Given the comparable mesh density and discretization strategy applied across all conventional and spherical models, this convergence result was considered representative of the mesh quality throughout the study.

## 4. Discussion

This study evaluated the effects of abutment–bar connection geometry, cantilever length, and bar prosthesis material on stress distribution in All-on-Four implant-supported prostheses with angled abutments. The results showed that increasing cantilever length increased stress levels in the prosthetic components and peri-implant bone tissues. This biomechanical expectation is consistent with the cantilever extension acting as a lever arm, and the bending moment increasing with distal extension length [38,39]. In the present study, the most pronounced stress concentrations were observed in the posterior region, particularly in the posterior angled abutment and posterior implant. This indicates that the distal part of the prosthetic assembly is the mechanically critical region, especially when extended cantilevers are used.

The spherical connection design reduced stress in the main posterior load-bearing components compared with the conventional design, especially under the 12 mm cantilever condition. This effect was more evident with the posterior-angled abutment and posterior implant, suggesting that modifying the abutment–bar interface geometry can alter the load-transfer pathway and reduce local stress accumulation. These results are consistent with previous studies reporting that connection geometry and prosthetic design details can influence stress distribution in implant-supported prostheses [27,29]. However, the results should not be overgeneralized. In the PEEK group, some anterior components, particularly the anterior bar prosthesis screw and anterior abutment, showed increased stress in the spherical models. This finding suggests that the spherical interface does not simply reduce stress throughout the entire prosthetic system; rather, it redistributes stress from the distal region to other components depending on material stiffness and contact behavior.

The highest stress value in the study was observed in the posterior angled abutment of the PC-12 model. Since the angled abutment was modeled as a titanium-based component, this value should be compared primarily with the mechanical strength of Ti6Al4V Grade 5 rather than with PEEK strength. The stress value of 1592.2 MPa in the posterior angled abutment of the PC-12 model exceeded the reported yield strength of Ti6Al4V Grade 5, indicating a potential mechanical risk for the abutment under the simulated loading condition [40]. In addition, the stress values calculated in the PEEK bar prosthesis exceeded the commonly reported strength limits for PEEK in some configurations, suggesting that PEEK may not be suitable as the sole bar-framework material in extended cantilever designs. This point matters because PEEK’s lower stiffness does not necessarily translate into improved mechanical safety for the entire prosthetic system.

The comparison between Ti6Al4V and PEEK bar prosthesis materials demonstrated that the elastic modulus of the framework material strongly affected the mechanical response of the whole system. Ti6Al4V provided a stiffer framework and generally limited deformation under distal loading. In contrast, PEEK, with a much lower elastic modulus, allowed greater elastic deformation of the bar prosthesis. Although this flexibility may reduce stress concentration within selected framework regions, it may also increase load transfer to screws, abutments, implants, and bone tissues. This interpretation is consistent with previous studies evaluating PEEK and related high-performance polymer frameworks for implant-supported prostheses [41]. Therefore, the biomechanical suitability of PEEK should not be evaluated only by considering stress within the bar prosthesis itself. A system-level interpretation is necessary because reduced framework stiffness may lead to unfavorable stress redistribution in other prosthetic components.

The peri-implant bone results also showed a material- and geometry-dependent response. The posterior cortical bone capsule exhibited higher stress values than the trabecular bone capsule in all model groups, which is consistent with the higher stiffness and load-bearing role of cortical bone. In the 12 mm cantilever models, the spherical design reduced posterior cortical bone stress compared with the conventional design in both Ti6Al4V and PEEK groups. Nevertheless, the cortical bone stress in the PC-12 conventional model was considerably higher than that in the corresponding Ti6Al4V model, indicating that the use of a low-stiffness framework material may increase bone-level stress under extended cantilever loading. Since excessive peri-implant bone stress has been associated with marginal bone loss and implant-related complications, load configuration and prosthetic material selection should be considered together during prosthetic planning [23,42].

From a clinical and biomechanical perspective, the findings support three main considerations. First, cantilever length should be minimized whenever anatomically and prosthetically feasible, because the 8 mm models generally produced lower stresses than the 10- and 12-mm models. Second, the spherical connection interface may reduce stress concentration in critical posterior components, particularly in extended cantilever configurations. Third, the use of PEEK as the sole bar framework material should be approached with caution in full-arch prostheses with long cantilevers, because its lower stiffness may increase stress transfer to screws, abutments, implants, and bone tissue. Therefore, the mechanical advantage of PEEK should not be evaluated only by the stress level within the bar prosthesis; system-level stress redistribution must also be considered.

Several limitations should be considered when interpreting the present findings. All materials were modeled as homogeneous, isotropic, and linearly elastic, although cortical and trabecular bone have anisotropic and heterogeneous mechanical properties in vivo [5,27]. Complete osseointegration was assumed at the implant–bone interface, and screw preload was not included in the model. The applied 100 N vertical load represented a simplified static loading condition and did not reproduce the multidirectional, cyclic, and patient-specific nature of mastication [30,42]. Mesh quality values were within an acceptable range according to previously reported criteria [31], and mesh independence of the reported peak stress value was further confirmed through a dedicated convergence analysis at the posterior angled abutment interface of the PC-12 model, where the highest stress was recorded (Table 5). In addition, the friction coefficients were assigned based on literature data rather than experimentally measured values for the exact component surfaces used in the model [32]. Therefore, the present results should be interpreted as comparative numerical findings rather than direct clinical predictions. Future studies should include screw preload, cyclic fatigue loading, anisotropic bone modeling, and experimental validation to confirm the mechanical reliability of the proposed spherical connection design.

This represents a reasonable and clinically relevant consideration, as posterior masticatory forces are rarely purely axial and commonly include oblique components. Previous finite element studies have shown that oblique loading generally amplifies and redistributes stress differently than axial loading. This effect can be particularly pronounced around angled abutments and across different implant–abutment connection designs [43,44]. For example, oblique loading has been reported to increase stress asymmetrically around angled abutments compared with axial loading, with the magnitude of the increase depending on the degree of abutment angulation [43,45]. Similarly, connection-design-dependent differences in stress distribution have been shown to persist, and in some cases intensify, under oblique loading relative to axial loading [44,46]. These findings suggest that the stress-reducing advantage of the spherical connection design observed in the present study under axial loading may not be fully generalizable to oblique or multidirectional masticatory conditions. A dedicated future study incorporating oblique loading vectors (e.g., 15° to 30° off-axis) is warranted to evaluate whether the spherical connection retains its advantage under such conditions.

## 5. Conclusions

Within the limitations of this finite element model, the results indicate that the mechanical behavior of bar-supported full-arch implant prostheses is governed not by a single design parameter, but by the combined effects of cantilever length, connection geometry, and framework material. The spherical abutment–bar interface provided a more favorable stress-transfer pattern in the distal load-bearing region, particularly around the posterior angled abutment and posterior implant, where the highest stress concentrations were observed. However, this design did not uniformly reduce stress across all components, especially when PEEK was used as the bar prosthesis material. Therefore, the spherical connection should be considered as a stress-redistribution design rather than a universally protective geometry. The findings also suggest that increasing cantilever length remains a critical biomechanical risk factor, and that PEEK, despite its elastic deformation capacity, may transfer higher stresses to prosthetic components and peri-implant bone under extended cantilever loading. From a design perspective, shorter cantilever extensions and Ti6Al4V bar frameworks appear to be mechanically more reliable under simulated static loading conditions, whereas using PEEK as the sole framework material should be approached with caution unless supported by further fatigue testing and experimental validation.

## Figures and Tables

**Table 1:** Node and element counts and mesh quality parameters of each model in the FEA.

Design of Dental Prosthetic Models	Nodes	Elements	Average Element Quality	Average Skewness
**C-8**	798,138	512,034	0.760 ± 0.204	0.323 ± 0.239
**C-10**	799,227	512,741	0.760 ± 0.203	0.323 ± 0.239
**C-12**	800,419	513,494	0.761 ± 0.203	0.323 ± 0.239
**S-8**	784,286	507,255	0.767 ± 0.201	0.314 ± 0.235
**S-10**	785,650	508,193	0.767 ± 0.201	0.315 ± 0.236
**S-12**	786,503	508,690	0.767 ± 0.201	0.314 ± 0.236

**Table 2:** Mechanical properties of the models used in the FEA.

Material Type	Poisson’s Ratios	Young’s Modulus (MPa)	References
**Ti6Al4V Grade 5** **(Density: 4.43 g/cm^3^)**	0.35	110.000	[18,28,29]
**PEEK** **(Density: 1.31 g/cm^3^)**	0.4	4100	[2,18]
**Cortical Bone** **(Density: 1.99 g/cm^3^)**	0.30	13.700	[28,29]
**Trabecular Bone** **(Density: 1.84 g/cm^3^)**	0.30	1.370	[28,29]

**Table 3:** Von Mises stress values in dental prosthesis components and bones (MPa)—bar prosthesis material: Ti6Al4V Grade 5.

	C-8	C-10	C-12	S-8	S-10	S-12
**Bar Prosthesis**	196.03	256.18	300.41	185.13	206.76	237.86
**Anterior Bar Prosthesis Screw**	66.48	87.05	108.29	58.08	72.35	86.70
**Anterior Abutment**	83.76	104.83	126.14	47.49	64.62	72.05
**Anterior Implant**	79.73	96.05	112.56	27.99	33.72	41.37
**Anterior Cortical Bone Capsule**	16.56	20.30	23.88	11.61	14.47	17.41
**Anterior Trabecular Bone Capsule**	1.85	2.37	2.89	1.55	1.91	2.27
**Posterior Bar Prosthesis Screw**	110.9	138.18	168.27	40.25	52.16	64.16
**Posterior Angled Abutment**	405.52	492.88	576.97	98.32	105.85	113.3
**Posterior Abutment-Implant Screw**	69.85	80.69	94.13	65.12	70.93	76.77
**Posterior Implant**	227.49	273.46	369.95	110	114.4	130.95
**Posterior Cortical Bone Capsule**	61.13	71.08	80.81	49.30	54	57.28
**Posterior Trabecular Bone Capsule**	6.86	7.37	7.50	6.36	6.92	7.16

**Table 4:** Von Mises stress values in dental prosthesis components and bones (MPa)—bar prosthesis material: PEEK.

	C-8	C-10	C-12	S-8	S-10	S-12
**Bar Prosthesis**	221.52	278.65	334.81	156.82	179.16	226.34
**Anterior Bar Prosthesis Screw**	65.93	81.72	97.52	363.04	452.25	552.73
**Anterior Abutment**	100.7	125.33	149.9	156.74	184.2	214.04
**Anterior Implant**	92.87	114.59	136.08	106.03	125.3	131.6
**Anterior Cortical Bone Capsule**	15.72	20.01	23.92	21.02	25.44	28.83
**Anterior Trabecular Bone Capsule**	1.31	1.58	1.84	2.15	2.70	3.24
**Posterior Bar Prosthesis Screw**	314.63	405.93	499.31	380.79	564.51	832.66
**Posterior Angled Abutment**	977.59	1303.1	1592.2	375.06	289.1	403.82
**Posterior Abutment-Implant Screw**	193.74	237.98	282.31	81.91	91.06	102.37
**Posterior Implant**	639.87	785	996.71	238.8	266.8	298.99
**Posterior Cortical Bone Capsule**	123.78	148.22	176.57	76.87	90.33	97.90
**Posterior Trabecular Bone Capsule**	10.65	13.01	15.22	8.93	9.60	9.17

**Table 5:** Mesh convergence analysis performed at the posterior angled abutment interface of the PC-12 model, the location of the highest von Mises stress recorded in the study.

Mesh Testing	Element Size (mm)	Elements	Nodes	Max. von Mises Stress (MPa)	% Change from Previous
**1 (Current PC12-Model)**	0.18	513,494	800,419	1592	---
**2**	0.17	520,768	811,188	1629	2.32%
**3**	0.16	529,400	823,915	1647	1.10%
**4**	0.15	539,683	838,889	1658	0.67%
**5 (Fine Mesh-PC12 Model)**	0.14	553,501	858,749	1664	0.36%

## Data Availability

The data that support the findings of this study are available from the corresponding author upon reasonable request.
